# Periodontal disease and the risk of prostate cancer: a meta-analysis of cohort studies

**DOI:** 10.1590/S1677-5538.IBJU.2020.0333

**Published:** 2021-01-20

**Authors:** Zhenlang Guo, Chiming Gu, Siyi Li, Shu Gan, Yuan Li, Songtao Xiang, Leiliang Gong, Shusheng Wang

**Affiliations:** 1 The Second Affiliated Hospital of Guangzhou University of Chinese Medicine Department of Urology Guangzhou China Department of Urology, The Second Affiliated Hospital of Guangzhou University of Chinese Medicine, Guangzhou, China; 2 National University of Singapore Department of mechanical engineering Kent Ridge Singapore Department of mechanical engineering, National University of Singapore, Kent Ridge, Singapore

**Keywords:** Periodontal Diseases, Prostatic Neoplasms, Systematic Review [Publication Type]

## Abstract

**Background:**

Periodontal disease is reportedly associated with the risk of various systemic diseases, including pancreatic and lung cancers. However, its association with prostate cancer remains inconclusive. Herein, we explored the association of periodontal disease with the risk of prostate cancer through a meta-analysis.

**Materials and Methods:**

MEDLINE, Embase, Web of Sciences and Cochrane Library databases were searched for eligible publications up to April 2020. Multivariate adjusted risk estimates with corresponding 95% confidence intervals (CIs) were extracted and calculated using random- or fixed-effect models.

**Results:**

Nine cohort studies involving 3.353 prostate cancer cases with 440.911 participants were identified and included in the meta-analysis. We found that periodontal disease significantly increased the risk of prostate cancer by 1.40-fold (hazard ratio [HR]=1.40, 95% CI: 1.16-1.70; P=0.001; I2=76.1%) compared with normal condition. Interestingly, the risk of developing prostate cancer was not significant in patients treated with periodontal therapy (HR=1.22, 95% CI: 0.86-1.73; P=0.272; I2=65.2%). The results of subgroup analyses were also consistent and significant when stratified by study design and follow-up period, whereas conflicting results were observed in periodontal disease ascertainment stratification. These findings were robust as indicated by sensitivity analyses.

**Conclusions:**

Periodontal disease was associated with the increased risk of prostate cancer, whereas no significant association was observed in patients treated with periodontal therapy. Hence, the awareness and importance for maintaining oral health should be improved, and the underlying mechanisms linking periodontal disease and prostate cancer should be fully explored in future research.

## INTRODUCTION

Prostate cancer is the most common cancer in men and the leading cause of cancer-related deaths worldwide (1, 2). Approximately 164.690 new cases of prostate cancer were diagnosed in 2019 and led to 29.430 deaths, as estimated by the American Cancer Society (3). Available data show that the risk factors for prostate cancer include age, family history and race, which limit its prevention (4, 5). Periodontal disease, a complex microbial inflammatory disease of the periodontium, partly causes tooth loss. The cumulative burden of periodontal disease increased significantly between 1990 and 2015, resulting in a 64% increase in disability, which posed a great public health challenge for policy makers (6). Moreover, severe periodontal disease has affected 743 million people worldwide (7). Recently, the association between periodontal disease and the risk of cancer development has attracted research attention, especially for pancreatic, head and neck, and lung cancers (8–10).

Results about the association between periodontal disease and prostate cancer are conflicting (11–13). For instance, Dizdar et al. (12) suggested that periodontal disease is not associated with the increased risk of prostate cancer (hazard ratio [HR]=3.75, 95% confidence interval [CI]: 0.95-10.21), whilst Arora et al. (11) and Guven et al. (13) reported different observations. Considering that a single epidemiological study may not be sufficient to determine the effect of periodontal disease on prostate cancer risk, we performed a systematic review and meta-analysis of previous studies to further elucidate the association between periodontal disease and prostate cancer risk.

## MATERIALS AND METHODS

This systematic review and meta-analysis were conducted in line with the Cochrane Collaboration criterion (14) and reported based on the Preferred Reporting Items for Systematic Reviews and Meta-Analyses (PRISMA) statement (Supplementary Material) (15).

### 

#### Search Strategy

MEDLINE (via PubMed), Embase, Web of Sciences and Cochrane Library databases were searched for eligible studies that investigated the association between periodontal disease and the risk of prostate cancer up to April 2020. The combination of Medical Subject Headings (MeSH) and non-MeSH terms was used in each database, including ‘periodontal disease’, ‘periodontitis’ or ‘dental health’ and ‘prostate carcinoma’, ‘prostate cancer’, ‘prostate neoplasms’ or ‘prostate tumor’, without restriction for language, region or publication status. The reference lists from previous reviews and other relevant articles were manually searched to identify additional studies. The main search was completed by the senior author (ZL Guo), and any discrepancy was resolved by consensus or consultation with another investigator (SS Wang).

#### Eligibility Criteria

The inclusion criteria for original studies that investigated the association between periodontal disease and prostate cancer risk were as follows: (1) studies reporting the risk estimate (HR, odds ratio [OR], relative risk, standardized incidence rate [SIR]) with associated 95% CIs of incident for prostate cancer (any stage) amongst participants with periodontal disease (i.e. periodontitis, tooth loss or gingivitis caused by periodontitis) compared with those free of periodontal disease; (2) the evaluation of periodontal status might vary across studies, including self-reporting, clinical diagnosis or retrieved from clinical and radiographic parameters; (3) observational studies (i.e. prospective or retrospective cohort, cross-sectional or case-control study) published as original articles; and (4) studies that provide sufficient raw data for calculation if no risk estimates with associated 95% CIs were reported. For studies on the same population or subpopulation, only the largest or most recent studies with the longest follow-up duration were considered. Case series, case reports and review articles were excluded. Disagreement was resolved through discussion amongst the investigators.

#### Data extraction and methodological quality assessment

The title and abstract of all articles retrieved from the initial search were screened to ascertain their relevance. All potentially relevant full-text articles were further considered and assessed to determine their inclusion eligibility in the meta-analysis. Hereafter, two investigators (CM Gu and SY Li) independently extracted and crosschecked the following data of all the included studies through a predesigned evidence table: first author, study population, study design, country, participant characteristics (i.e. sample size and age), follow-up duration, periodontal disease ascertainment, therapy of periodontal disease, dental and smoking status, risk estimates with associated 95% CIs or sufficient raw data and corresponding adjusting confounding factors. If the information in the included studies was insufficient, the primary author was contacted to obtain and verify the data.

The methodological quality of the included studies was assessed by two investigators (S Gan and Y Li) on the basis of the Newcastle-Ottawa scale (NOS) (16), which includes nine items that assess the representativeness of eligible studies. In detail, the evaluation of each item could be classified as ‘unclear’, ‘yes’ or ‘no’, which corresponded to ‘0’, ‘1’ or ‘0’, respectively. The total score ranged from 0 to 9, where 8-9 indicates high quality, 6-7 indicates moderate quality and ≤5 indicates low quality. Any disagreement was resolved by consensus or consultation with a third investigator.

### Statistical Analysis

The association between periodontal disease and prostate cancer was evaluated using risk estimates, and their corresponding 95% CIs were extracted from the included studies via Stata version 15.0 (serial number: 10699393; StataCorp Wyb). For consistent definitions, the differences amongst the various measures of risk estimates could be ignored because periodontal disease-related prostate cancer is a rare event; therefore, the ORs and SIRs were directly considered as HRs in the meta-analysis (17). I2 was used to investigate heterogeneity amongst the included studies, and high statistical heterogeneity was defined as I2 ≥50%. High heterogeneity warrants the use of random-effect inverse-variance models; otherwise, a fixed-effect model should be utilized (15). Statistical significance was considered at P <0.05. Subgroup analyses stratified by study design, follow-up duration, country, periodontal disease ascertainment and therapy of periodontal disease were performed. Sensitivity analysis was also performed by deleting each study individually to assess the stability and consistency of results. Meta-regression analysis was performed to investigate the potential risk factors of heterogeneity, and restricted maximum likelihood was used in the analysis. However, the use of Egger (18) and Begg-Mazumdar (19) tests was limited because of the limited number of studies evaluated.

## RESULTS

### 

#### Study identification and selection

**Figure 1:**
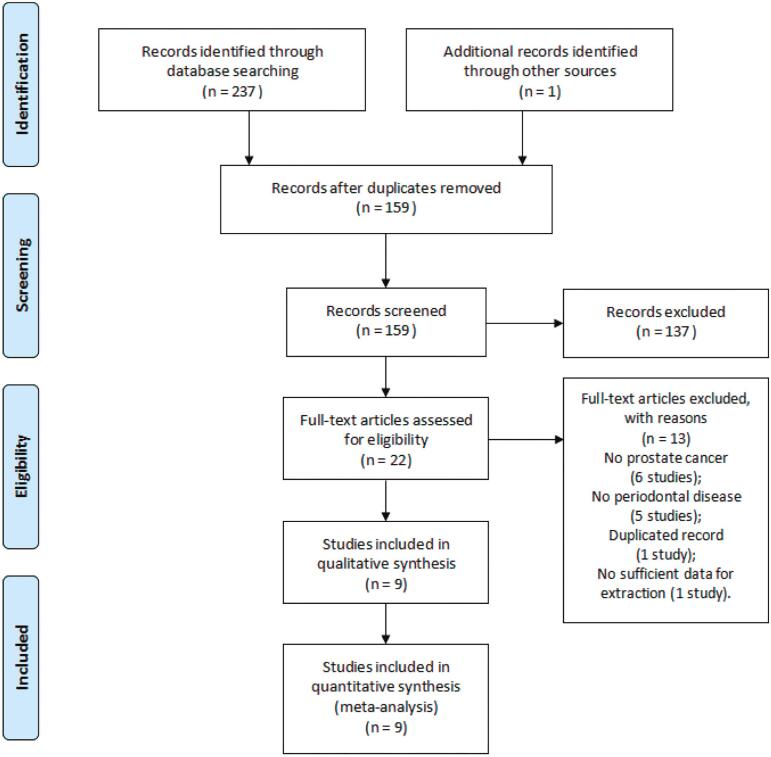
Flow diagram of literature searches according to the Preferred Reporting Items for Systematic Reviews and Meta-Analyses statement.

Figure-1 presents the search and study selection process. Overall, the initial search yielded a total of 238 articles. After duplication, the titles and abstracts of 159 articles were evaluated for eligibility, where 22 full-text articles were retrieved for further evaluation and 13 were excluded for the following reasons: no prostate cancer (6 studies), no periodontal disease (5 studies), duplication (1 study) and no sufficient data for extraction (1 study). Nine articles (11-13, 20-25) involving 3.353 prostate cancer cases with 440.911 participants were identified and included in the meta-analysis according to the eligibility criteria.

#### Study characteristics and methodological quality

The main characteristics of the included studies are summarized in Table-1. These studies included four prospective cohorts (11, 20, 23, 24) and five retrospective cohorts (12, 13, 21, 22, 25), which were published from 2003 to 2019. Three studies (20, 23, 24) from the United States, two (12, 13) from Turkey, two (21, 25) from Taiwan, one (11) from Sweden and one (22) from Korea were included. Moreover, the sample sizes varied from 1.250 and 187.934, and the follow-up period ranged from 7.2 years to 27 years. However, three studies did not provide information on the follow-up duration (21, 22, 25). Regarding the ascertainment of periodontal disease, three studies (11, 23, 24) used a self-reporting method, three studies (20, 21, 25) adopted a clinical diagnosis method, and three studies (12, 13, 22) used clinical radiographic parameters. Moreover, three studies (12, 24, 25) reported patients with periodontal disease and a history of periodontal treatment or currently undergoing periodontal therapy, whereas six studies (11, 13, 20–23) did not provide this information. One study (20) focused on the association between gingivitis and prostate cancer risk, and another study (22) provided information on the baseline smoking status of participants. Notably, all the included studies reported risk estimates adjusted for confounding factors.

**Table 1:** Characteristics of the included studies.

First author year	Study population	Study design (Duration)	Country	Sample size (PCa cases)	Age (years)	Follow-up period (years)	PD ascertainment	PD therapy	Adjustments	Dental/smoking status (severity)
Arora, et al. 2010 (11)	The Swedish Twin Registry	Prospective cohort (1963-2004)	Sweden	15.333 (604)	38-77	27	Self-report	Unreported	Age, education, employment, number of siblings, smoking status, smoking status of partner, alcohol status, body mass index, and diabetes	PD
Dizdar, et al. 2017 (12)	The Hacettepe University Dentistry and Oncology hospitals in Ankara	Retrospective cohort (2001-2010)	Turkey	1.250 (3 cases with CP)	49.6	12	Clinical and radiographic parameters	Included	Age	CP (moderate or severe)
Güven, et al. 2019 (13)	The Hacettepe University Dentistry and Oncology hospitals in Ankara	Retrospective cohort (2007-2012)	Turkey	5.199 (40 cases with PD)	57.7	7.2	Clinical and radiographic parameters	Unreported	Age	PD
Hujoel, et al. 2003 (20)	The NHANES I Epidemiologic Follow-up Study (NHEFS)	Prospective cohort (1971/1975-1992)	USA	11.328 (67)	25-74	10	Clinical diagnosis	Unreported	Age, poverty index, education, race, smoking, vitamin A and C consumption, and alcohol consumption	Periodontitis Gingivitis
Hwang, et al. 2014 (21)	Taiwan National Health Insurance (NHI) system	Retrospective cohort (1996-2010)	Taiwan	38.902 (250 cases with PD)	43.1±13.6	NA	Clinical diagnosis	Unreported	Age, occupation, type 2 diabetes mellitus, hypertension, and hyperlipidemia	PD
Lee, et al. 2017 (22)	National Health Insurance Service-Health Examinee Cohort (NHIS-HEC)	Retrospective cohort (2002-2013)	Korea	187.934 (934)	≥40	NA	Clinical and radiographic parameters	Unreported	Age, household income, insurance status, residence area, hypertension, diabetes mellitus, cerebral infarction, angina pectoris, myocardial infarction, smoking status, alcohol intake, and regular exercise	PD Current smokers
Michaud, et al. 2016 (23)	The Health Professionals Follow-up Study (HPFS)	Prospective cohort (1986-2012)	USA	19.933 (696)	40-75	26	Self-report	Unreported	Age, race, alcohol use, physical activity, history of diabetes, body mass index, geographical location, height, and NSAID use	PD
Michaud, et al. 2018 (24)	Atherosclerosis Risk in Communities study cohort (ARIC)	Prospective cohort (1987-2012)	USA	7.466 (375)	44-66	14.7	Self-report	Included	Age, field center, education level, smoking status, smoking duration, drinking status, body mass index, and diabetes status	Periodontitis (Moderate)
Wen, et al. 2014 (25)	Taiwan National Health Insurance (NHI) system	Retrospective cohort (1997-2010)	Taiwan	153.566 (384)	45.2± 14.8	NA	Clinical diagnosis	Included	Age, diabetes, hypertension and hyperlipidemia	PD

**CP** = chronic periodontitis; **NA** = not applicable; **NSAID** = nonsteroidal anti-inflammatory drug; **PCa** = prostate cancer; **PD** = periodontal disease

In general, the quality of the included studies (11–13, 20–25) was methodologically evaluated on the basis of NOS. Four studies (11, 20, 23, 24) acquired scores of 8 or 9 and were considered as high quality; four studies (13, 21, 22, 25) acquired scores of 6 or 7 and were considered as moderate quality; one study (12) obtained a score of 5 and was considered as low quality.

#### Periodontal disease and prostate cancer risk

**Figure 2:**
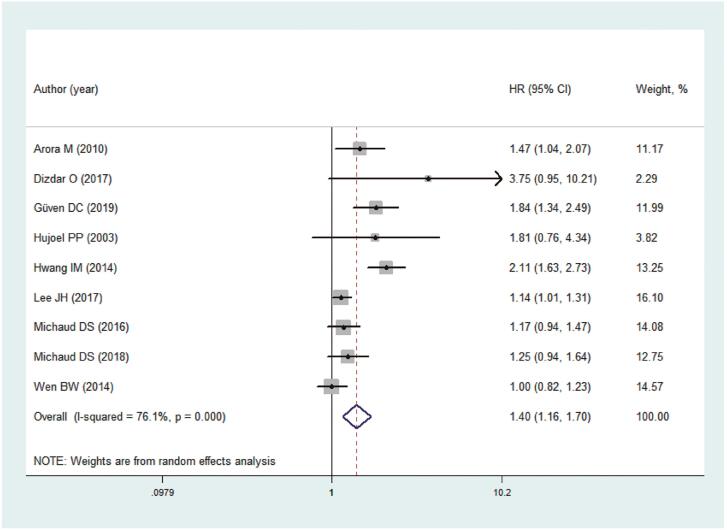
Meta-analysis on association between periodontal disease and prostate cancer risk. CI, confidence interval, HR, Hazard Ratio.

The results of meta-analysis revealed that periodontal disease significantly increased the risk of developing prostate cancer by 1.40 times (HR=1.40, 95% CI: 1.16-1.70; P=0.001; I2=76.1%), and the risk was greater than those without periodontal disease. Significant statistical heterogeneity was observed. Thus, we used a random-effect model (Figure-2). Interestingly, the risk of developing prostate cancer was not significant in patients treated with periodontal therapy (HR=1.22, 95% CI: 0.86-1.73; P=0.272; I2=65.2%) compared with those who have never been treated for periodontal disease (HR=1.49, 95% CI: 1.17-1.91; P=0.001; I2=79.2%). In the subgroup analyses of study design, excess risk of prostate cancer was observed in prospective (HR=1.27, 95% CI: 1.09-1.48; P=0.003; I2=0%) and retrospective cohorts (HR=1.51, 95% CI: 1.09-2.09; P=0.013; I2=87.3%). When stratified by follow-up period, two cohorts reporting a follow-up period of more than 15 years suggested that periodontal disease can increase the risk of prostate cancer (HR=1.26, 95% CI: 1.02-1.56; P=0.030; I2=15.8%), thereby supporting the results of four cohorts with a follow-up period of less than 15 years (HR=1.64, 95% CI: 1.17-2.29; P=0.004; I2=47.5%). Regarding the diagnosis of periodontal disease, the pooled results of three studies indicated significant association based on the self-reported methods (HR=1.25, 95% CI: 1.07-1.46; P=0.005; I2=0%), whereas negative results were observed based on the other two methods (Table-2). We were unable to perform a subgroup analysis based on the smoking status of the study participants and the severity of periodontal disease because of limited data.

**Table 2:** Results of subgroup analyses.

Overall results		Studies, N	Participants,N	HR (95% CI)	p value	p of heterogeneity	I2 (%)
		9	440.911	1.40 (1.16-1.70)	0.001	<0.001	76.1
**Study design**	
	Prospective cohort	4	54.060	1.27 (1.09-1.48)	0.003	0.603	0
	retrospective cohort	5	386.851	1.51 (1.09-2.09)	0.013	<0.001	87.3
**Country**	
	Sweden	1	15.333	1.47 (1.04-2.07)	0.028	NA	NA
	Turkey	2	6.449	2.07 (1.23-3.46)	0.006	0.255	22.7
	USA	3	38.727	1.22 (1.03-1.45)	0.023	0.621	0
	Taiwan	2	192.468	1.45 (0.70-3.01)	0.323	<0.001	95
	Korea	1	187.934	1.14 (1.00-1.30)	0.048	NA	NA
**PD therapy**	
	Included	3	162.282	1.22 (0.86-1.73)	0.272	0.056	65.2
	Unreported	6	278.629	1.49 (1.17-1.91)	0.001	<0.001	79.2
**PD ascertainment**	
	Self-report	3	42.732	1.25 (1.07-1.46)	0.005	0.552	0
	Clinical and radiographic parameters	3	194.383	1.61 (0.99-2.61)	0.056	0.004	82.1
	Clinical diagnosis	3	203.796	1.52 (0.83-2.79)	0.177	<0.001	90.2
**Follow-up period**	
	>15 years	2	35.266	1.26 (1.02-1.56)	0.030	0.276	15.8
	<15 years	4	25.243	1.64 (1.17-2.29)	0.004	0.126	47.5
	NA	3	380.402	1.32 (0.92-1.92)	0.136	<0.001	90.9

**CI** = confidence interval; **HR** = Hazard Ratio; **NA** = not applicable; **PD** = periodontal disease

Sensitivity analysis validated the stability of our results by omitting every study. Notably, the results of meta-regression analyses indicated that the variables (study design, P=0.573, R-squared [R2] values=-8.67%; country, P=0.281, R2=-11.64%; follow-up period, P=0.915, R2=-12.07%; periodontal disease ascertainment, P=0.583, R2=-7.34%; periodontal disease therapy, P=0.686, R2=-4.10%) could not result in heterogeneity amongst the included studies. Moreover, the adjusted R2 values from −12.07% to −4.10% revealed that these regressors slightly contributed to the explanation of the response variables.

## DISCUSSION

### 

#### Main findings

The association between periodontal disease and prostate cancer was assessed using nine pooled studies, which involved 3.353 prostate cancer cases amongst 440.911 participants. The results suggest that periodontal disease is associated with increased risk of prostate cancer. Interestingly, the risk of developing prostate cancer was not significant in patients treated with periodontal therapy. Moreover, the results of subgroup analyses were consistent and significant when stratified by study design and follow-up period, whereas conflicting results were observed in periodontal disease ascertainment stratification. The results were robust as indicated by the sensitivity analysis. However, the meta-regression failed to identify the potential confounding factors that might affect the level of heterogeneity amongst the included studies.

Several studies shared conflicting results of the association between periodontal disease and prostate cancer (20–22). In a prospective cohort with 67 prostate cancer cases amongst 11.328 participants, Hujoel et al. (20) reported a negative association between periodontitis and prostate cancer risk (OR=1.81, 95% CI: 0.76-4.34) and gingivitis (OR=1.48, 95% CI: 0.56-3.94). By contrast, Hwang et al. (21) and Lee et al. (22) demonstrated that periodontal disease is associated with the excess risk of prostate cancer. Lee et al. focused on the influence of smoking status on prostate cancer risk amongst patients with periodontal disease and revealed that current smokers with periodontal disease had a significantly increased risk of prostate cancer, that is, 1.68 times (HR=1.68, 95% CI: 1.52-1.85) greater than that for non-smokers. However, our understanding of the effect of smoking status and the severity of periodontal disease on prostate cancer risk remains insufficient because of the limited studies evaluated.

Notably, a prospective cohort regarding the topic was excluded from the meta-analysis because it did not meet the inclusion criteria. We found that Michaud et al. performed two prospective cohorts in 2016 (23) and 2008 (26) based on a similar population from the same database. The cohort comprising 541 prostate cancer cases with 48.375 participants was collected from the Health Professionals Follow-up Study database performed by Michaud et al. (26). It revealed that periodontal disease is not significantly associated with the increased risk of prostate cancer (HR=0.90, 95% CI: 0.73-1.12). To avoid duplication, we identified cohorts with more prostate cancer cases and more comprehensive information according to the inclusion criteria. Notably, the results of the sensitivity analysis and meta-regression further consolidated our findings.

#### Comparison with previous study

A meta-analysis regarding the association between periodontitis and cancer risk was published by Corbella et al. (27). Although the main results of our meta-analysis were consistent with those in previous studies, several differences between the results of Corbella et al. and the current work should be noted. Firstly, Corbella et al. only included two studies comprising 1.237 prostate cancer cases with 68.308 participants. By contrast, our meta-analysis included nine cohorts involving 3.353 prostate cancer cases with 440.911 participants. With the added statistical power of seven studies and at least 2116 prostate cancer cases with 372.603 participants, our meta-analysis was the latest and the most comprehensive review to date. Secondly, contrary to the study of Corbella et al., the current meta-analysis excluded a cohort performed by Michaud et al. (26) to avoid duplication. Finally, meta-regression and subgroup analyses stratified by study design, country, follow-up period, periodontal disease ascertainment and periodontal disease therapy were also performed to identify the potential risk factors that might affect the level of heterogeneity amongst the included studies. Moreover, sensitivity analysis reinforced the main findings of our meta-analysis.

#### Implications for clinical practice

The incidence of prostate cancer is increasing year by year with the improvement of prostate biopsy technology (28, 29). However, the etiological relationships between periodontal disease and prostate cancer remain controversial, and little is known about their underlying mechanisms. Hence, further studies on the pathogenesis of prostate cancer and clinical and epidemiological evidence are urgently needed to explore the relationships between periodontal disease and prostate cancer. Given the rising prevalence of periodontal disease worldwide, if the underlying mechanism is confirmed, this observation will be beneficial for clinicians and public health decision makers in the management of prostate cancer. Amongst the included studies, several trials used self-reported methods as an ascertainment of periodontal disease. However, any misclassification would underestimate the association between periodontal disease and prostate cancer. Periodontal disease may be worsened in patients with osteoporosis because of androgen deprivation therapy (ADT). Famili et al. (30) found that patients diagnosed with prostate cancer receiving ADT developed periodontal disease compared with those who did not receive ADT. Therefore, clinicians must consider this observation in patients receiving ADT. As the etiology of prostate cancer develops, increasing evidence suggests that chronic or recurrent inflammation may also be associated with prostate cancer risk. Moreover, the low level of persistent systemic inflammation caused by periodontal disease can induce oxidative DNA damage, uncontrolled repair procedures and eventually the occurrence of tumors in the body (31–33). Therefore, if a genetic link can be determined between periodontal disease and prostate cancer, the specific identification of genetic polymorphism may be beneficial to identifying high-risk groups and developing preventive strategies, which merits further attention. Finally, for patients with periodontal disease, a high-risk group for developing prostate cancer, increased awareness and effective periodontal therapy should be immediately applied by clinicians to reduce the risk of developing prostate cancer. Notably, patients with prostate cancer should be encouraged to pay more attention to their own oral health care, and urological clinicians and nurses should provide oral health-care tips, education, etc. to better manage patients with prostate cancer.

#### Strengths and limitations

Overall, our study exhibited several crucial strengths. Firstly, it was the latest and most comprehensive meta-analysis regarding the association between periodontal disease and the risk of prostate cancer. Moreover, subgroup analyses stratified by study design, country, follow-up period, periodontal disease ascertainment and periodontal disease therapy were performed to determine whether these variables affected the level of heterogeneity amongst the included studies. Secondly, all the risk estimates extracted from the included studies were adjusted for confounding factors to minimize their effect on the overall results. Finally, sensitivity analysis and meta-regression further validated and reinforced the rationality and reliability of our findings.

However, the meta-analysis was restricted by several limitations. Firstly, five studies used retrospective cohort design, which might miss data and result in a risk of bias. Secondly, significant heterogeneity was observed. Moreover, none of the variables that might affect the level of heterogeneity were identified although sensitivity analysis revealed the robustness of the overall results. Finally, we were unable to further investigate the association between smoking status and the degree of severity of periodontal disease because of the limited data. Therefore, future high-quality research should comprehensively address these issues.

## CONCLUSIONS

Existing evidence suggests that periodontal disease appears to be associated with an increased risk of developing prostate cancer. Interestingly, no significant association was observed in patients who underwent periodontal therapy. Hence, patients with periodontal disease, a high-risk group for developing prostate cancer, should be treated with periodontal therapy immediately. Furthermore, the awareness and importance of maintaining oral health should be improved based on the main findings. Notably, further research should fully explore the underlying mechanisms linking periodontal disease and prostate cancer.
